# Influence of Alternating Multi-Layered Design on Damping Characteristics of Butyl Rubber Composites and a New Idea for Achieving Wide Temperature Range and High Damping Performance

**DOI:** 10.3390/polym14245484

**Published:** 2022-12-15

**Authors:** Chao Qin, Qiang Feng, Jie Zhang, Jiang Li, Shaoyun Guo

**Affiliations:** State Key Laboratory of Polymer Materials Engineering, Polymer Research Institute, Sichuan University, Chengdu 610065, China

**Keywords:** butyl rubber, alternating multi-layered, free damping, constrained damping, micro-constrained

## Abstract

This paper investigates the influence of an alternating multi-layered design on the material loss factor and effective temperature range of free/constrained-damping butyl rubber, and then proposes a new method of designing materials with high damping properties and a wide temperature range. First, the wide-temperature rubber IIR-0, the low-temperature rubber IIR-1, the medium-temperature rubber IIR-2, and the high-temperature rubber IIR-3 are prepared and characterized. Second, the influences of an alternating multi-layered design on the damping peak values and temperature range of free damping and micro-constrained damping of the rubber types are investigated. Finally, different methods for broadening the damping temperature range and improving the damping loss factor are discussed. The results show that the loss factor of the alternating multi-layered, constrained damping structure is increased to 0.488, while that of the free-damping structure is increased to 0.845. Their damping-temperature ranges are increased to 89.4 °C and 93.2 °C, respectively. A wide temperature range and high damping performance can be achieved by the alternating multi-layered design of rubber/plastic micro-constrained damping composites.

## 1. Introduction

Noise and vibration performance are the core indicators of equipment. Viscoelastic polymers are the key material to reducing vibration and controlling noise [1]. The operating environments of different types of equipment are complex. For example, high-speed trains in China operate across the north and south, while ships cruise in different areas of the sea. They are faced with seasonal changes with a relatively large range of temperature changes. Therefore, when developing damping materials, we should not only increase their damping loss factors but also broaden their effective damping temperature ranges [2].

However, most rubbers without modification have low glass transition temperatures and a narrow effective damping temperature range [3]. To overcome this defect, polymers should usually be copolymerized, incorporate an Interpenetrating Polymer Network (IPN), be blended, etc. Suresh et al. [4] prepared a butyl acrylate/acrylonitrile copolymer. The damping properties increased with the increase in the mass fraction of butyl acrylate in the copolymer. Patri et al. [5] prepared interpenetrating polymer networks (IPNs) of styrene butadiene rubber (SBR) and poly (alkyl methacrylate). The damping temperature range became wider due to micro-heterogeneous phase separation. The synthesis process of IPN and copolymerization are complex and expensive, which limits the applications of damping modification materials [6]. Among them, blending modification is the most convenient and effective method for improving damping performance. Liang et al. [7] and Wu et al. [8] prepared a rubber blend with petroleum resin. The damping peak moved to a high temperature and the damping temperature range expanded with the increase in the resin content. Wan et al. [9] found that, with the increase in the C9 petroleum resin content in PBMA, the damping peak increased to a higher temperature and the range of effective damping temperature was widened. Zhao et al. [10] and Wang et al. [11] blended AO-60 with nitrile butadiene rubber. With the increase in the amount of AO-60, the enhancement of the degree of hydrogen bonding between AO-60 and the NBR matrix led to a shift in the damping peak temperature from low to high and the broadening of the damping temperature range. Kongsinlark et al. [12] added monodisperse polyisoprene-SiO_2_ nanoparticles to natural rubber. They found that the damping loss factor decreased significantly and the glass transition temperature moved towards a high temperature. Li et al. [13] found that the addition of petroleum resin in CIIR could greatly improve a material’s damping factor and broaden its damping range. Sheng et al. [14] added polysiloxane microspheres to CIIR composites. After blending, the damping temperature range in the high-temperature region was broadened. Qu et al. [15] added phenolic resin to chlorinated butyl rubber to achieve blending modification and Hu et al. [16] added C5 petroleum resin to the ion-modified, brominated butyl rubber BIIR. They all found that the damping temperature range widened while the damping peak gradually decreased with the increase in resin content. Wu et al. and Shen et al. [17,18] found that, with the increase in the petroleum resin content, the damping loss peaks of the CIIR/P70 blend system and the IIR/HDCPD blend system all moved towards high temperatures, and the damping peak increased while the damping temperature range narrowed. Li et al. [19] blended acrylic rubber (ACM), polylactic acid (PLA), and phenolic resin to prepare damping materials. Consequently, it was found that the peak value of the materials’ loss factor became higher, and the damping temperature range widened.

In addition to the improvement to the damping properties of materials via blending, copolymerizing, and IPN, there are also many studies focusing on the layered design of damping materials. For example, some research [20,21] shows that the damping loss peak of a multi-layered structure increases with the number of layers. The shear strain is mainly distributed on the damping layer, and the more constrained the damping layers are, the larger the average shear strain is. Therefore, a multi-layered design has a positive effect on the damping performance of polymers. Zhang et al. [22] prepared CIIR/PVC alternating multi-layer damping composites. Due to their multi-layered structures, the two damping loss peaks of rubber and plastic partially overlapped. The damping properties of the multi-layer damping composites were better than those of traditional blend composites. As the number of layers increased, the effective damping temperature range gradually broadened. Hiltern et al. [23,24] prepared thousands of alternating polymer nano-layered structures. When the limited polyethylene oxide (PEO) layer thickness was reduced from the micron level to the nanometer level, the crystal morphology changed from three-dimensional spherulites to lamellar structures parallel to the multilayer interface. Ding et al. [25] designed a new multilayer, chlorinated polyethylene. This organic hybrid material had different damping loss peaks and glass transition temperatures with different amounts of 2,2′- methylene bis-(4-methyl-6-cyclohexyl) phenol. With more layers of damping materials, multi-layered damping materials with wide and high damping ranges could be obtained.

At present, this field mainly focuses on simultaneously increasing the damping factor and widening the damping temperature range. Due to the uncertainty of the influencing factors in the material-blending or layered design process, there are deficiencies in the process of improving damping performance, such as the weakening of the damping peak, the contraction of the effective temperature range, and so on. If we can first prepare a damping material with a broad temperature range, and then increase its damping peak via a micro-constrained alternating layered design, we can produce a new method for the preparation of high-damping performance and wide-damping temperature range materials. Therefore, based on the existing research results, this paper conducts an innovative research according to this assumption. We first investigate the influence of an alternating multi-layered design on the material loss factor and effective temperature range of free/constrained damping butyl rubber, and then propose a new way to design materials with high damping properties and a wide temperature range. Firstly, butyl rubber (IIR) was modified with petroleum resin (P125) and polyisobutylene (PIB). Low-temperature rubber IIR-1, medium-temperature rubber IIR-2, and high-temperature rubber IIR-3 were also prepared. IIR-0 with a wide temperature range was prepared via the damping-based modification of butyl rubber with expanded graphite. Secondly, taking the IIR-2 as a sample, the influences of the alternating multi-layered design on the damping loss factor and the temperature range of the free-damping and constrained-damping materials were investigated. Finally, the performances of the alternating layered design of low/high-temperature rubber with free-damping properties and the alternating layered design of wide-temperature range rubber/plastics with micro-constrained damping properties were studied and compared. Compared to the alternating layered design of low/high-temperature rubber-based free damping, the wide-temperature range rubber/plastics with micro-constrained damping can avoid uncertainty with respect to the material’s temperature range and damping matching. Therefore, a more controllable temperature range and damping design can be obtained.

## 2. Materials and Methods

### 2.1. Materials

IIR (BK1675N), with an isoprene concentration of 1.6%, a density of 0.92 g/cm^3^ and a Mooney viscosity of 51 (ML1+8, 125 °C), was purchased from Nizhnekamsk (Russia). Hydrogenated petroleum resin(P125), with a softening point of 125 °C, was purchased from Idemitsu Kosan (Tokyo Metropolis, Japan). Polyisobutylene (PIB-2400), with an average molecular weight of 2400, was supplied by Korean Daelim Co., Ltd. (Seoul, Republic of Korea). Polymethyl methacrylate (PMMA) brand CM-211, with a density of 1.19 g/cm^3^, was produced by Jiangsu Zhenjiang Qimei Co., Ltd. (Zhenjiang, China). CaCO_3_, with a density of 2.71 g/cm^3^, was supplied by TianWei Industry Co., Ltd. (Chengdu, China). Expanded graphite (EG250) was produced by Sungraf Materials Group Limited (Qingdao, China). Carbon black (N550) was produced by Cabot (Boston, MA, USA).

### 2.2. Instruments and Characterization

The morphological structure of alternating layered composites was studied by a polarizing microscope (Nikon POM, LV100POL, made in Japan). The frozen ultra-thin slicer was used to slice the cross-sectional direction of the alternating laminated composite sample to obtain the samples with a regular surface layered structure. The samples with the regular surface layered structure were placed on the polarizing microscope platform, and the layered structure of the samples was observed by using the multiple of the eyepiece (10 times) × objective (5 times). Dynamic mechanical analysis (DMA) was obtained using a dynamic mechanical analyzer (Q800, TA instrument). The samples with a length of 34 mm, a width of 13 mm, and a height of 3 mm were heated from −80 °C to 100 °C at a heating rate of 3 °C/min. The test mode was single cantilever beam mode, the dynamic strain was 0.04%, the test frequency was 10 Hz, and the dynamic load direction was perpendicular to the interface.

### 2.3. Sample Preparation

The short chain of polyisobutylene will rub with the molecular chain of butyl rubber internally, converting more mechanical energy into internal energy, and improving the damping effect [26]. Calcium carbonate plays a role in filling material volume and reducing cost. Adding petroleum resin (P125) is an effective method to adjust the damping peak temperature. Adding expanded graphite is an effective method to broaden the damping temperature range. So, IIR, PIB-2400, and CaCO_3_ are all designed as 100 phr.

We expect to prepare three high-damping materials with the same damping peak temperature difference (about 40 °C) at low temperature, medium temperature, and high temperature, respectively. The higher the content of P125, the higher the damping peak temperature [27]. Therefore, the composites IIR-1/2/3 with different damping peak temperatures can be obtained by adjusting the content of P125, while the damping composite IIR-0 with a wide temperature range can be obtained by adding expanded graphite [28]. So, P125 and EG are designed as different components.

#### 2.3.1. Preparation of Modified IIR

First, the kneader is preheated at 130 °C for 10 min, and butyl rubber is cut and put into the kneader for stirring for 30 min. After the butyl rubber is kneaded into fine particles, PIB-2400 and CaCO_3_ are added. Then, P125 is added according to the formula in Table 1 for a 90-min kneading. Subsequently, the addition of expanded graphite needs to be operated in an open smelting machine at 35 °C.

#### 2.3.2. Preparation of Alternating Multi-Layered Structure

**Figure 1:**
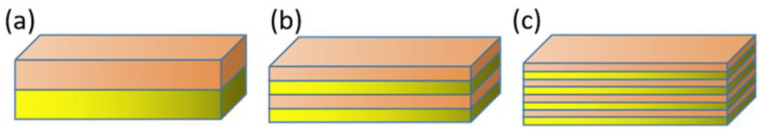
Schematic diagram of IIR-2/PMMA multi-layered micro-constrained damping structures: (**a**) 2 layers; (**b**) 4 layers; and (**c**) 8 layers.

Four kinds of modified IIR are made into three kinds of thin sheets of 1.5 mm, 0.75 mm, and 0.375 mm, respectively, by press moulding and heating at 80 °C. PMMA is pressed into 1.5 mm, 0.75 mm, and 0.375 mm thin sheets by press moulding and heating at 180 °C. Then, samples with different thicknesses are made into alternating layered free-damping structures and micro-constrained damping structures with 2, 4, and 8 layers with a total thickness of 3 mm, by press moulding and heating at 80 °C. A schematic diagram of the layered structures is shown in Figure 1. For example, IIR-2/PMMA-2/4/8 layered micro-constrained damping structures are composed of 1.5 mm IIR-2 and 1.5 mm PMMA, 2 layers of 0.75 mm IIR2 and 2 layers of 0.75 mm PMMA, and 4 layers of 0.375 mm IIR-2 and 4 layers of 0.375 mm PMMA, respectively.

## 3. Results and Discussion

### 3.1. Dynamic Mechanical Properties of IIR-0/IIR-1/IIR-2/IIR-3

**Figure 2:**
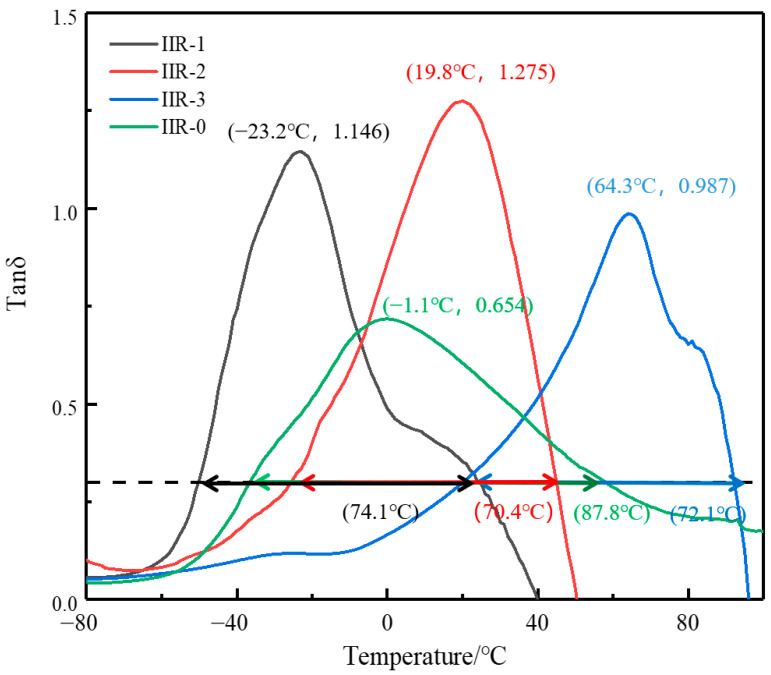
The temperature dependence of tanδ at 10 Hz for four kinds of IIR.

The low-temperature rubber IIR-1, medium-temperature rubber IIR-2, and high-temperature rubber IIR-3 were prepared by adjusting the content of P125 petroleum resin. At the same time, the IIR-0 with a wide temperature range was prepared by damping modification of butyl rubber with expanded graphite. The temperature range of the IIR-0 is significantly larger than that of the IIR-1, IIR-2, and IIR-3. The damping properties of the four damping butyl rubber were studied using DMA. Figure 2 shows the change of loss factor of the four kinds of damping butyl rubber with temperature. It can be seen from Figure 2 that the damping loss peaks of the IIR-1, IIR-2, and IIR-3 are 1.146, 1.275, and 0.987, respectively. The corresponding peak damping temperatures are −23.2 °C, 19.8 °C, and 64.3 °C, respectively. The effective temperature ranges are 74.1 °C, 70.4 °C, and 72.1 °C, respectively. The addition of low molecular weight P125 petroleum resin limits the movement of butyl rubber molecular chain, leading to the rise of damping loss peak temperature. Therefore, we successfully prepared three kinds of butyl rubber with a damping peak temperature interval of about 40 °C and a damping temperature range of about 70 °C. The damping peak value of the IIR0 is 0.654, the damping peak temperature is −1.1 °C, and the damping temperature range is 87.8 °C. This is because the addition of flake graphite improves the micro-constrained damping structure of the material.

### 3.2. Effect of Alternate Layered Design on Free Damping

**Figure 3:**
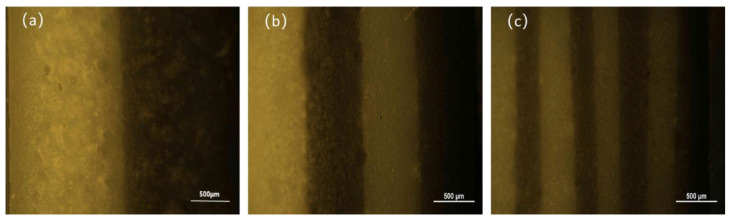
Polarized light micrograph images of the IIR-2 with (**a**) 2 layers; (**b**) 4 layers; and (**c**) 8 layers.

The medium-temperature rubber IIR-2 is divided into thin sheets of 1.5 mm, 0.75 mm, and 0.375 mm according to the thickness of the rubber layer. Composite structures with a total thickness of 3 mm are made by alternating lamination of thin sheets. The layered arrangement is shown in Table 2. In order to observe the alternating layered structure of butyl rubber, 0.5% carbon black was added to the IIR-2 damping butyl rubber for dyeing. Polarization microscopic micrographs of two, four, and eight layers of the IIR-2 damping rubber materials are shown in Figure 3.

**Figure 4:**
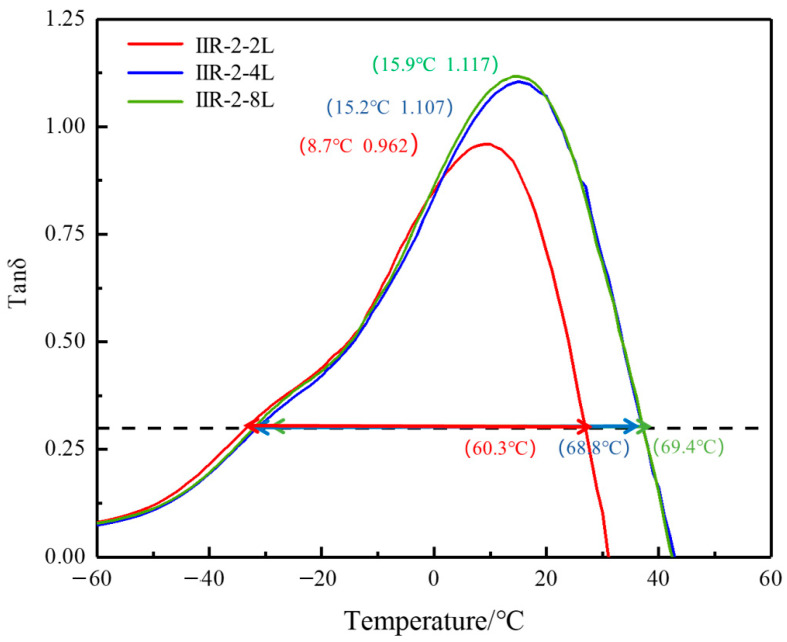
The temperature dependence of tanδ at 10 Hz for IIR-2 with different layers.

Figure 4 shows the change in the loss factor of two, four, and eight layers of the IIR-2 with temperature. The damping peak value, the damping peak temperature, and the damping temperature range of the multilayer composites are shown in Figure 4. With the increase in the number of alternate layers, the damping peak value gradually increases from 0.962 to 1.117. At the same time, the damping peak moves to the high temperature region, and the peak temperature from low to high is 8.7 °C, 15.2 °C, and 15.9 °C.

The damping loss factor increases with the number of layers. This is because the existence of a layered structure will lead to shear strain at the layer interface, and the shear action of molecular chains at the layer interface will convert more mechanical energy into heat energy. The shear strain increases with the number of alternate layers, so the more layers, the better the damping effect. However, the difference between the damping peak value and damping temperature range of 4- and 8-layered structures is small.

### 3.3. Effect of Alternate Layered Design on Micro-Constrained Damping

**Figure 5:**
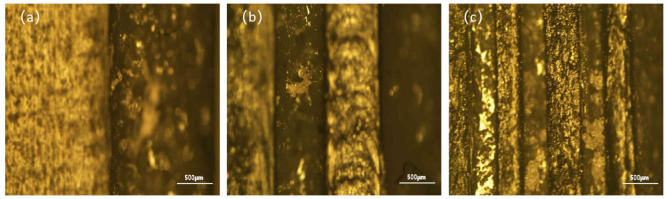
Polarized light micrograph of the IIR-2/PMMA with different layers: (**a**) 2 layers; (**b**) 4 layers; and (**c**) 8 layers.

The IIR-2 and PMMA of 1.5 mm, 0.75 mm, and 0.375 mm are alternately laminated to make the micro-constrained damping structures. The total thickness of the micro-constrained damping structures is 3 mm, including 2, 4, and 8 layers, respectively. The layered arrangement is shown in Table 3. We explored the influence of different layers on the damping performance of the layered damping materials. The polarizing microscopic micrograph of the two, four, and eight layers of the IIR-2/PMMA alternating layered composites is shown in Figure 5.

**Figure 6:**
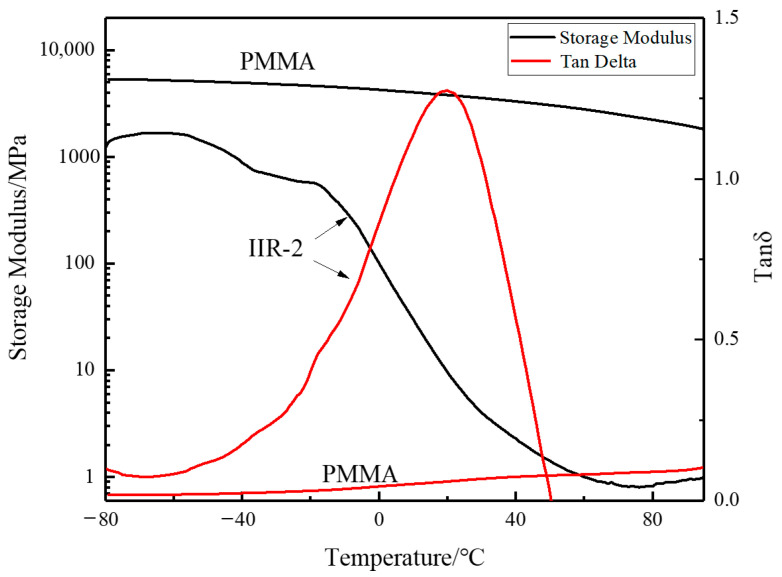
Temperature dependence of storage modulus and tanδ of PMMA and IIR-2.

Figure 6 shows the changes in storage modulus and damping loss factor of the IIR-2 and PMMA with temperature. It can be seen from Figure 6 that the storage modulus of the PMMA is always greater than that of the IIR-2 in the temperature range of −80 °C to 80 °C. With the temperature rising from −60 °C to 40 °C, the storage modulus of the IIR-2 decreases rapidly, while that of the PMMA decreases slowly. In the temperature range from −80 °C to 80 °C, the PMMA damping loss factor is very small and increases slightly. The damping loss factor of the IIR-2 is significantly greater than the PMMA. It can reach the damping peak at 20 °C. At this time, it exhibits a high tanδ peak (1.275) and a wide temperature range of 70.4 °C.

**Figure 7:**
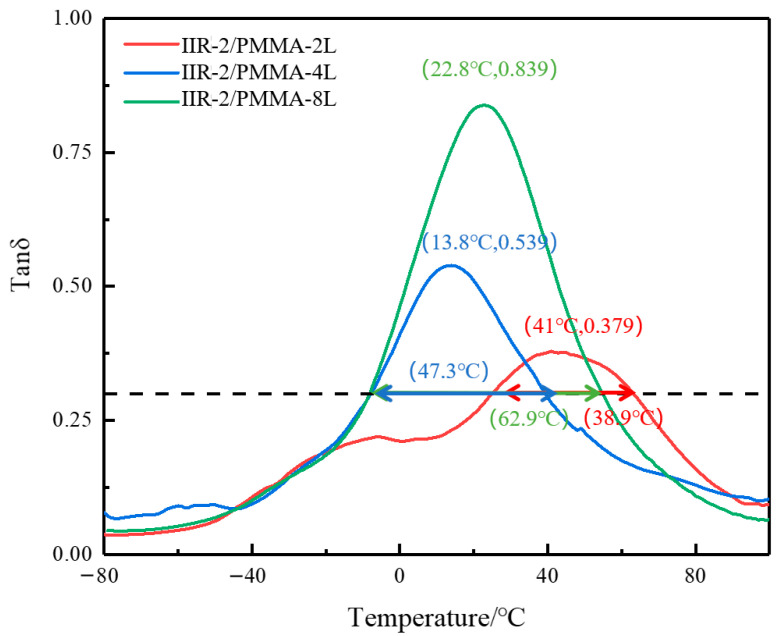
The temperature dependence of tanδ at 10 Hz for IIR-2/PMMA with different layers.

Figure 7 shows the change in damping loss factor with temperature for the 2-, 4-, and 8-layered IIR-2/PMMA alternating layered composites. It can be observed that the peak of the damping loss factor increases with the number of layers. The peak of the 8-layered rubber/plastic alternating layered composites is much larger than that of 2-layered composites. The damping peak increases from 0.3791 to 0.8387, with a growth rate of 121.2%. With the increase in the number of layers, the damping performance of the rubber/plastic alternating layered composites significantly improves at about 20 °C. Therefore, the micro-constrained damping structure formed by laminated rubber and plastic is an effective way to improve the damping of two-phase materials, especially to increase the peak damping loss factor.

The IIR-2 of rubber system has poor compatibility with the PMMA of plastic system. They have different molecular structures, large differences in polarity, large differences in free volume between molecular chains, and poor mutual viscosity between two systems. The glass transition temperature (about 110 °C) of the PMMA is much higher than the IIR-2. The damping loss factor of the PMMA is very small at room temperature. Due to their weak interaction, the PMMA will not interfere with the segment relaxation behaviour of the IIR-2. That means the damping peak temperature of the rubber/plastic alternating layered composites is mostly near room temperature. With the increase in the number of layers, more layered interfaces are formed. The interfacial friction between the layers will produce shear action, which dissipates more energy. Therefore, the more layers, the better the damping effect. Compared to the damping performance of the IIR-2 free damping alternating layer, it can be seen that the damping of the alternating layered structure with micro-constrained damping of different systems is more obviously affected by the number of layers.

### 3.4. Different Methods for Broadening the Damping Temperature Range and Improving the Damping Loss Factor

As mentioned above, on the one hand, the research of damping materials should obtain a high damping peak for a specific temperature. On the other hand, it is expected to obtain a wide enough damping temperature range. A commonly used method to improve the damping peak and broaden the temperature range is to use rubber with different temperature ranges for multi-layered design. In order to verify the new method envisaged in this paper, namely first prepare damping materials with a certain temperature range through material modification and then improve the damping peak based on micro-constrained alternating multi-layered design, the effect of layers on the damping temperature range of the low-temperature rubber/high-temperature rubber alternating layered composites and the broad temperature range rubber/plastic alternating layered composites are compared and studied.

#### 3.4.1. Alternating Layered Design of Low/High-Temperature Rubber Free Damping

**Figure 8:**
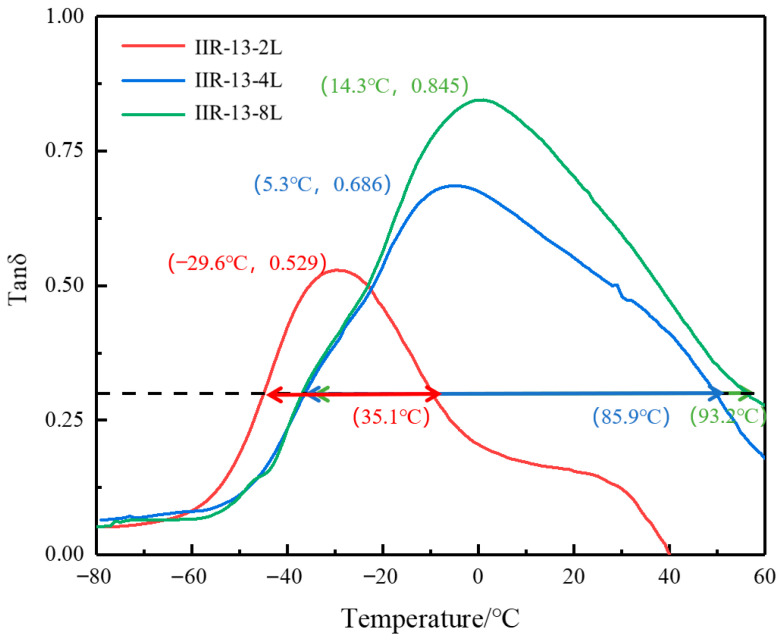
The temperature dependence of tanδ at 10 Hz for IIR-1/IIR-3 with different layers.

Figure 8 shows the change in damping loss factor of the two, four, and eight layers of the low-temperature rubber IIR-1 and the high-temperature rubber IIR-3 with temperature. It can be seen that each of the three alternating layered composites has only one loss damping peak. With the increase in the number of layers, the damping loss factor becomes larger and the temperature range becomes wider. We can see that the maximum damping loss factor increases from 0.529 to 0.845, and the damping temperature range increases from 35.1 to 93.2 °C. This is because the damping butyl rubber with different glass transition temperatures under the same system has formed a macroscopic incompatible system of two consecutive phases due to the laminated structure, and each has formed a continuous phase. With the increase in the number of layers, the number of two-phase interface and phase domain increases. Therefore, the purpose of improving damping peak and broadening temperature range can be achieved by preparing damping materials with different temperature ranges and performing an alternating layered design.

#### 3.4.2. Alternating Layered Design of Wide Temperature Range Rubber/Plastics with Micro-Constrained Damping

**Figure 9:**
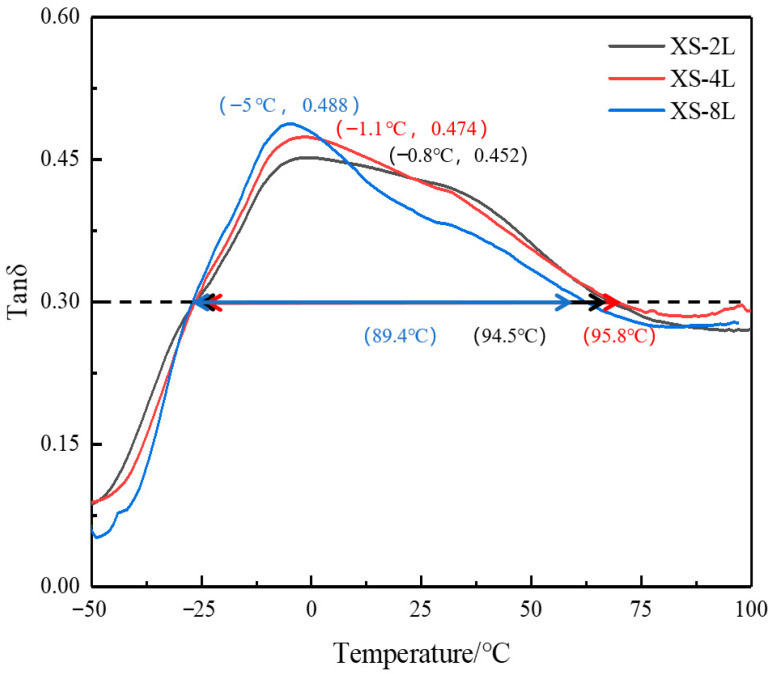
The temperature dependence of tanδ at 10 Hz for IIR-0/PMMA with different layers.

Figure 9 shows the change in damping loss factor with the temperature of the PMMA/IIR-0 alternating layered composites, which are stacked in two, four, and eight layers according to the thickness ratio of 1:4. As shown in Figure 9, each of the three alternating layered composites has only one loss peak. With the increase in the number of layers, the temperature range of the alternating layered composites does not change significantly. However, the damping loss peak increases from 0.452 to 0.488 with the number of layers. The maximum damping loss factor of a constrained damping structure with eight layers is 8% higher than that of the two-layered structure. This is because the constrained damping structure formed by the IIR0 and PMMA has interlaminar shear action. The more layers, the stronger the interlaminar shear action. The damping effect becomes better. Therefore, we can prepare damping materials with a certain temperature range by material modification. Then, the damping peak can be increased based on micro-constrained structure and alternating layered design. Finally, a broad temperature range and high damping peak material is prepared. However, the damping peak value in Figure 9 is not high enough compared with Figure 8. It should be related to the material selection of the constraint layer, the layer thickness ratio of the constraint layer, and the damping layer, which need to be further optimized in the future.

For the multi-layered free damping [20,25,29] and multi-layered rubber/plastic constrained damping [21,22,30], their damping temperature ranges are generally about 50~129 °C, and their damping loss factors are generally about 0.4~1.8. In this paper, the alternating multi-layered free damping has a loss factor of 0.845 and a temperature range of 93.2 °C. While the alternating multi-layered constrained damping has a loss factor of 0.488 and a temperature range of 89.4 °C. Therefore, both of the two damping composites present good damping loss factor and temperature range.

## 4. Conclusions

(1) Low-temperature rubber (IIR-1, −50.6 °C~23.5 °C, tanδ > 0.3), medium-temperature rubber (IIR-2, −25.1 °C~45.3 °C, tanδ > 0.3) and high-temperature rubber (IIR-3, 20.1 °C~92 °C, tanδ > 0.3) with a relatively same temperature range (72 ± 2 °C) are prepared by adjusting the content of P125 petroleum resin. A wide-temperature-range rubber (IIR-0, −33 °C~54.8 °C, tanδ > 0.3) is prepared by damping modification of butyl rubber with expanded graphite.
(2) For the alternating multi-layered micro-constrained damping, the loss factor of the 8-layered composites is 2.21 times higher than that of the 2-layered composites. While for the alternating multi-layered free damping, the loss factor of the 8-layered composites is 1.6 times higher than that of the 2-layered composites. Alternating multi-layered design can improve the damping loss factor of composites.
(3) The alternating multi-layered free damping has a loss factor of 0.845 and a temperature range of 93.2 °C, while the alternating multi-layered constrained damping has a loss factor of 0.488 and a temperature range of 89.4 °C. It is effective and feasible to obtain high damping and wide temperature range by first preparing damping materials with a wide temperature range, and then improving the damping peak based on micro-constrained alternating layered design.

## Figures and Tables

**Table 1:** Sample preparation formulae for IIR-0, IIR-1, IIR-2, and IIR-3.

Sample	IIR	PIB-2400	CaCO_3_	P125	EG
IIR-0	100	100	100	70	80
IIR-1	100	100	100	/	/
IIR-2	100	100	100	80	/
IIR-3	100	100	100	240	/

**Table 2:** IIR-2 layered structure.

Sample	Layered Structure
IIR-2-2L	IIR-2/IIR-2
IIR-2-4L	IIR-2/IIR-2/IIR-2/IIR-2
IIR-2-8L	IIR-2/IIR-2/IIR-2/IIR-2/IIR-2/IIR-2/IIR-2/IIR-2

**Table 3:** IIR-2/PMMA layered structure.

Sample	Layered Arrangement
IIR-2/PMMA-2L	IIR-2/PMMA
IIR-2/PMMA-4L	IIR-2/PMMA/IIR-2/PMMA
IIR-2/PMMA-8L	IIR-2/PMMA/IIR-2/PMMA/IIR-2/PMMA/IIR-2/PMMA

## Data Availability

The data presented in this study are available from the corresponding author upon request.

## References

[1] Ammineni S.P., Nagaraju C., Raju D.L. (2022). Modal performance degradation of naturally aged NBR. Polym. Test..

[2] Cui H.W., Jing Q., Li D.W., Zhuang T.T., Gao Y.X., Ran X.H. (2023). Study on the high-temperature damping properties of silicone rubber modified by boron-terminated polysiloxane. J. Appl. Polym. Sci..

[3] Chen B.W., Dai J.W., Song T.S., Guan Q.S. (2022). Research and Development of High-Performance High-Damping Rubber Materials for High-Damping Rubber Isolation Bearings: A Review. Polymers.

[4] Suresh K.I., Sitaramam B.S., Raju K. (2003). Effect of copolymer composition on the dynamic mechanical and thermal behaviour of butyl acrylate-acrylonitrile copolymers. Macromol. Mater. Eng..

[5] Patri M., Reddy C.V., Narasimhan C., Samui A.B. (2007). Sequential interpenetrating polymer network based on styrene butadiene rubber and polyalkyl methacrylates. J. Appl. Polym. Sci..

[6] Lei T., Zhang Y.W., Kuang D.L., Yang Y.R. (2019). Preparation and Properties of Rubber Blends for High-Damping-Isolation Bearings. Polymers.

[7] Liang J.Y., Chang S.Q., Feng N. (2013). Effect of C5 petroleum resin content on damping behavior, morphology, and mechanical properties of BIIR/BR vulcanizates. J. Appl. Polym. Sci..

[8] Wu C.Y., Wu G.Z., Wu C.F. (2006). Dynamic mechanical properties in blends of poly(styrene-b-isoprene-b-styrene) with aromatic hydrocarbon resin. J. Appl. Polym. Sci..

[9] Wan S.H., Zhou S.S., Huang X., Chen S.B., Cai S.W., He X.R., Zhang R. (2020). Effect of Aromatic Petroleum Resin on Damping Properties of Polybutyl Methacrylate. Polymers.

[10] Zhao X.-Y., Cao Y.-J., Zou H., Li J., Zhang L.-Q. (2012). Structure and dynamic properties of nitrile-butadiene rubber/hindered phenol composites. J. Appl. Polym. Sci..

[11] Wang X., Chen X., Song M., Wang Q., Zheng W., Song H., Fan Z., Myat Thu A. (2020). Effects of Hindered Phenol Organic Molecules on Enhancing Thermo-Oxidative Resistance and Damping Capacity for Nitrile Butadiene Rubber: Insights from Experiments and Molecular Simulation. Ind. Eng. Chem. Res..

[12] Kongsinlark A., Rempel G.L., Prasassarakich P. (2012). Synthesis of monodispersed polyisoprene-silica nanoparticles via differential microemulsion polymerization and mechanical properties of polyisoprene nanocomposite. Chem. Eng. J..

[13] Li C., Wu G., Xiao F., Wu C. (2007). Damping behavior of sandwich beam laminated with CHR/petroleum resins blends by DMA measurement. J. Appl. Polym. Sci..

[14] Sheng Z.Y., Wang J.C., Yang S.Y., Song S.Q. (2019). Novel polysiloxane microspheres: Preparation and application in chlorinated butyl rubber (CIIR) damping composites. Adv. Powder Technol..

[15] Qu L., Huang G., Wu J., Tang Z. (2007). Damping mechanism of chlorobutyl rubber and phenolic resin vulcanized blends. J. Mater. Sci..

[16] Hu X., Zhang R.N., Wemyss A.M., Du A.H., Bao X.J., Geng X.Y., Wan C.Y. (2022). Damping and Electromechanical Behavior of Ionic-Modified Brominated Poly(isobutylene-co-isoprene) Rubber Containing Petroleum Resin C5. Ind. Eng. Chem. Res..

[17] Li C., Xu S.-A., Xiao F.-Y., Wu C.-F. (2006). Dynamic mechanical properties of chlorinated butyl rubber blends. Eur. Polym. J..

[18] Shen M.L., Xia L.C., Feng Q., Zhang J., Li J., Guo S.Y. (2021). Damping characteristics of a multi-layered constrained beam using viscoelastic butyl rubber layer with wide temperature range. Mater. Express.

[19] Li C.L., Ji X.X., Lyu Y., Shi X.Y. (2019). A strengthening approach for damping property and shape memory property of acrylic rubber/polylactide blends. J. Elastomers Plast..

[20] Yang H.L., Zhang W., Moffitt R.D., Ward T.C., Dillard D.A. (2007). Multi-layer in-situ for evaluation of dynamic mechanical properties of pressure sensitive adhesives. Int. J. Adhes. Adhes..

[21] Zhang F.S., Guo M.L., Xu K.M., He G.S., Wu H., Guo S.Y. (2014). Multi-layered damping composites with damping layer/constraining layer prepared by a novel method. Compos. Sci. Technol..

[22] Zhang F.S., He G.S., Xu K.M., Wu H., Guo S.Y. (2015). The Damping and Flame-Retardant Properties of Poly(vinyl chloride)/Chlorinated Butyl Rubber Multi-layered Composites. J. Appl. Polym. Sci..

[23] Wang H.P., Keum J.K., Hiltner A., Baer E., Freeman B., Rozanski A., Galeski A. (2009). Confined Crystallization of Polyethylene Oxide in Nanolayer Assemblies. Science.

[24] Lai C., Ayyer R., Hiltner A., Baer E. (2010). Effect of confinement on the relaxation behavior of poly(ethylene oxide). Polymer.

[25] Ding X.B., Liu T., Zhang Y.H., Han J. (2012). Analysis and prediction of damping properties of multi-layered organic hybrid materials consisting of polarized polymers and small molecules. Advanced Materials Research.

[26] Xia L.C., Li C.H., Zhang X.M., Wang J.F., Wu H., Guo S.Y. (2018). Effect of chain length of polyisobutylene oligomers on the molecular motion modes of butyl rubber: Damping property. Polymer.

[27] Yun Y.M., Lee J.H., Choi M.C., Kim J.W., Kang H.M., Bae J.W. (2021). A Study on the Effect of Petroleum Resin on Vibration Damping Characteristics of Natural Rubber Composites. Elastomers Compos..

[28] Fang L.H., Shen Z., Li J.F., Huang A.H., Lin M.S., Su Z.Z. (2022). Damping properties of expanded graphite filled fluorinated polyacrylate composites. Polym. Bull..

[29] Qin R., Huang R.L., Lu X. (2018). Use of gradient laminating to prepare NR/ENR composites with excellent damping performance. Mater. Des..

[30] Xu K.M., Hu Q.M., Wang J.H., Zhou H.D., Chen J.L. (2019). Towards a Stable and High-Performance Hindered Phenol/Polymer-Based Damping Material Through Structure Optimization and Damping Mechanism Revelation. Polymers.

